# QsrO a Novel Regulator of Quorum-Sensing and Virulence in *Pseudomonas aeruginosa*

**DOI:** 10.1371/journal.pone.0087814

**Published:** 2014-02-13

**Authors:** Thilo Köhler, Hajer Ouertatani-Sakouhi, Pierre Cosson, Christian van Delden

**Affiliations:** 1 Departement of Microbiology and Molecular Medicine, University of Geneva, Geneva, Switzerland; 2 Department of Physiology and Metabolism, University of Geneva, Geneva, Switzerland; 3 Service of Infectious Diseases, University Hospitals Geneva, Geneva, Switzerland; National Institutes of Health, United States of America

## Abstract

In *Pseudomonas aeruginosa*, the production of many secreted virulence factors is controlled by a quorum-sensing (QS) circuit, constituted of transcriptional activators (LasR, RhlR, PqsR) and their cognate signaling molecules (3-oxo-C12-HSL, C4-HSL, PQS). QS is a cooperative behavior that is beneficial to a population but can be exploited by “QS-cheaters”, individuals which do not respond to the QS-signal, but can use public goods produced by QS-cooperators. In order to identify QS-deficient clones we designed a genetic screening based on a *lasB*-*lacZ* fusion. We isolated one clone (PT1617) deficient in QS-dependent gene expression and virulence factor production despite wild type *lasR*, *rhlR* and *pqsR* alleles. Whole genome sequencing of PT1617 revealed a 3,552 bp deletion encompassing ORFs PA2228-PA2229-PA2230 and the *pslA* gene. However, complementation of PT1617 by plasmid-encoded copies of these ORFs, did not restore QS. Unexpectedly, gene expression levels of ORFs PA2228, PA2227 (*vqsM*) and PA2222, located adjacent to the deletion, were 10 to 100 fold higher in mutant PT1617 than in PAO1. When expressed from a constitutive promoter on a plasmid, PA2226, alone was found to be sufficient to confer a QS-negative phenotype on PAO1 as well as on PA14. Co-expression of PA2226 and PA2225 in PAO1 further prevented induction of the type III secretion system. In summary, we have identified a novel genetic locus including ORF2226 termed *qsrO* (QS-repressing ORF), capable of down-regulating all three known QS-systems in *P. aeruginosa*.

## Introduction

Quorum-sensing (QS) is a widespread regulatory mechanism among bacteria to coordinate in a population the expression of important cellular functions, including the production of virulence determinants [1]. QS is a cooperative behaviour and as such is vulnerable to exploitation by QS-deficient mutants, so-called cheaters [2]. Such mutants gain a temporary fitness advantage when growing under QS-requiring conditions, since they benefit from public goods, generally extracellular products generated by QS-proficient clones, without paying the cost for their production [3].

QS has been extensively studied in the opportunistic pathogen *Pseudomonas aeruginosa*, where it involves three regulatory networks each consisting of one transcriptional regulator (LasR, RhlR or PqsR) and its associated signalling molecule (3-oxo-C12-HSL, C4-HSL and Pseudomonas Quinolone Signal (PQS), respectively) [4]. When cell density increases, the signalling molecules bind to their cognate transcriptional regulators and activate in a coordinated manner the expression of different target gene repertoires.

LasR mutants can be considered as social cheaters and have been shown to appear both under defined *in vitro* conditions [3], [5], [5,6], as well as *in vivo* in infected mice [7] and in patients colonized by *P. aeruginosa* [8]–[10].

According to microarray experiments performed under defined laboratory conditions, the *las* system controls the expression of more than 150 target genes [11], [12]. The *las*-controlled repertoire partially overlaps with the *rhl* and *pqs*-controlled repertoires due to a hierarchical architecture. This hierarchy however is modulated by environmental conditions and growth substrates [13]. The *las* system is itself under the control of the local repressor gene *rsaL* [14] as well as peripheral regulators including Vfr [15], QscR [16], [17], VqsR [18], VqsM [19] and the small RNA binding protein RsmA [20].

Apart from *lasR*, this complex regulatory network presents several other potential candidates which mutation could lead to a QS-deficient phenotype, as suggested recently by the occurrence of a QS-deficient *pqsR* cheater mutants when *P. aeruginosa* was grown on caseinate as the sole C-source [21]. In an attempt to identify new elements in this complex regulatory network, we used here a genetic screen to select for QS-deficient mutants.

## Materials and Methods

### Bacterial Strains and Media

Bacterial strains used are listed in Table 1. Strains were grown in Luria-Bertani (LB) medium at 37°C with agitation (220 rpm) or in PB (Difco Peptone) buffered with M9 salts medium without NH_4_Cl [22]. For pyocyanin determination strains were grown in glycerol-alanine medium (per liter: 6 g DL-alanine, glycerol 1% (vol/vol), 2 g MgSO_4_, 0.1 g K_2_HPO_4_, 0.1 g ferric citrate).

**Table 1:** Bacterial strains, plasmids and primers.

Strains plasmids primers	Relevant characteristics	Reference, source
*E. coli*		
DH10B	F– *mcr*A Δ(*mrr*-*hsd*RMS-*mcr*BC) Φ80*lac*ZΔM15 Δ*lac*X74	Laboratory
	*rec*A1 *end*A1 *ara*D139 Δ(*ara leu*)7697 *gal*U *gal*K *rps*L *nup*G	collection
S17-1λpir	Tp^R^ Sm^R^, recA, thi, pro, *hsd*R-M^+^RP4∶2-Tc:Mu: Km Tn7 λpir	Laboratory
		collection
*P. aeruginosa*		
PAO1	PT5, Wild type	Laboratory
		collection
PA14	Wild type	[38]
PAO1	AHQ biosensor strain, CTX *pqsA*::*luxCDABE*	[36]
*pqsA*::*lux*		
PAO1-W5	3.5 kbp deletion in PA2228*-pslA* region, pTS400	This study
PT1617	PAO-W5 after spontaneous loss of pTS400	This study
PT466	PAO1Δ*lasI*::Tc	[22]
PT498	PAO1Δ*lasR*::Tc	[22]
PT1850	PAO1-UW *vqsM*::Tn*5*-Tc	[39]
PT1833	PAO1-UW *pqsA*::Tn*5-*Tc	[39]
PT1842	PAO1Δ*vqsM*	This study
**Plasmids**		
pEX18T	Suicide vector; Tet	[47]
pRK2013	RK2, helper plasmid; Km	[28]
pSB1075	*lasR*, *lasI*-*luxCDABE*, ColE1; Ap	[33]
pTS400	*lasB*-*lacZ* translational fusion; Ap	[35]
pEX1.8	expression vector, lacI^Q^; Ap	[48]
pJPP8	pEX1.8, ptac::rhlR; Ap	[48]
pIApX2	pUCP20 derivative containing promoter pX2 and gfp; Ap	Ina Attree
pLIGF1	*lasI*-*gfp* promoter fusion; Ap	This study
ppqsA1	*pqsA*-*gfp* promoter fusion; Ap	This study
pME3872	*lasR* on mini-Tn7 gene delivery vector; Ap Gm	[25]
pME3078	Mobilizable suicide vector, ColE1; Tc	[27]
pHO1.1	1,383 bp PCR fragment harboring PA2228, cloned in *Bam*HI-	This study
	*Hin*dIII cleaved pIApX2; Ap	
pHO2.1	1,483 bp PCR fragment harboring PA2227 (vqsM) cloned in	This study
	*Bam*HI-*Hin*dIII cleaved pIApX2; Ap	
pHO3.1	1,698 bp PCR fragment harboring PA2229-PA2230 cloned in	This study
	*Bam*HI-*Hin*dIII cleaved pIApX2; Ap	
pHO4.1	6,023 bp PCR fragment harboring PA2227-PA2222 cloned in	This study
	*Bam*HI-*Hin*dIII cleaved pIApX2, Ap	
pHO6.1	4,059 bp DNA fragment harboring PA2227-PA2225, obtained	This study
	by *Xba*I digest and religation of pHO4.1; Ap	
pHO9.1	2,000 bp PCR fragment harboring PA2226-PA2225 cloned in	This study
	*Bam*HI-*Hin*dIII cleaved pIApX2; Ap	
pHO12.1	646 bp PCR fragment harboring PA2225 cloned in *Bam*HI-	This study
	*Hin*dIII cleaved pIApX2; Ap	
pHO13.1	765 bp PCR fragment harboring PA2226- cloned in *Bam*HI-	This study
	*Hin*dIII cleaved pIApX2; Ap	
**Primers**	5′- 3′ sequence	
PA2225-Bam	AGCTGGATCCAGATCAGGGTGGCGTCAGAGTA	This study
PA2225-Hind	AGCTAAGCTTCATTGAGCATGAGCTGGAAAA	This study
PA2226-Bam	AGCTGGATCCGAGCAATTGCACTGCTACTGAA	This study
PA2226-Hind	AGCTAAGCTTCCGATCAGACTCAGCAATACCA	This study
PA2228-Bam	CAGTGGATCCTGAAATGCCTTGATCTTCACC	This study
PA2228-Hind	CAGTAAGCTTGGCGCCATTCAAAATCAAATA	This study
PA2228-Bam2	CAGTGGATCCAGGGATATGCCTGGCTCATTT	This study
PA2222-Hind	CAGTAAGCTTGAACTTGTGCGCATATTTGG	This study
vqsM-Bam	CAGTGGATCCTGAGTGGGTCAAGGCATCAAC	This study
vqsM-Hind	CAGTAAGCTTCGCTAATTAGTCCAGCAAGCA	This study
PA2229-Bam	CAGTGGATCCGTAAGCACCGTAGCGTGCAA	This study
PA2230-Hind	CAGTAAGCTTCTCGTCGAAGGCGGAAGAG	This study
PA2228-1	GTATGGGCCCCTCACAGTTTA	This study
PA2228-2	CGACCGCCATGAAGAAATAAT	This study
PA2222-1	ATGCAATCCTGGCTTTGTCTTC	This study
PA2222-2	AACGTTGCAAACACGTTCCTTT	This study
vqsM-1	CGTCAGCGAATCGAGTTATTCC	This study
vqsM-2	TCTTTGCGGGCCTTATCTACAA	This study
PA0439-Bgl	CGCAGATCTATCCAGGTCGACGGACAGATG	This study
PA0439-Hind	GCGAAGCTTGTAGTCGAAGGCGGGTCGAT	This study
vqsM-upR-Gm	TCAGAGCGCTTTTGAAGCTAATTCGGCGTCATTCCACTC	This study
	TGGCTTA	
vqsM-dnF-Gm	AGGAACTTCAAGATCCCCAATTCGCGTCAGCGAATCGAG	This study
	TTATTCC	
ppqsA-Hind	ACACAAGCTTGATTTCAACAGGGAAGCCTGC	This study
ppqsA-Xba	ACACTCTAGAGAAATCGAGGCGGAACAGAAC	This study

### DNA Manipulations

Primers used for amplification of target genes were generated using the primer3 program (http://bioinfo.ut.ee/primer3-0.4.0/primer3/) and are listed in Table 1. Bacterial cell lysates were used as DNA templates for all PCR-amplifications. PCR conditions were as follows: denaturation at 95°C for 2 min, followed by 27 cycles of 95°C for 20 s, 57°C for 30 s, 72°C for 1 min/kbp and a final extension at 72°C for 4 min. The *lasI*-*gfp* fusion plasmid pLIGF1 was constructed by cloning a 780 bp *Bgl*II-*Eco*RV fragment from plasmid pBBR1-GFP [23] into *Bam*HI-*Eco*RV cleaved plasmid pPCS223 containing a *lasI*-*lacZ* transcriptional fusion [24]. A *pqsA*-*gfp* fusion was constructed by amplifying a 560 bp DNA fragment from PAO1 harbouring the *pqsA* promoter region flanked by *Hin*dIII and *Xba*I restriction sites and subsequent ligation into *Hin*dIII-*Xba*I cleaved plasmid pLIGF1, yielding plasmid ppqsA1. Plasmids were transferred into *P. aeruginosa* by electroporation. The miniT7 plasmid pME3872, harbouring the *lasR* gene was transferred by conjugation into *P. aeruginosa* as described previously [25].

Whole genome sequencing on strain PT1617 was performed by Illumina Sequencing (Fasteris SA, Plan-les-Ouates, Switzerland). Contigs were aligned on the genome sequence of PAO1 (NC_002516, www.pseudomonas.com).

### VqsM (PA2227) Mutant Construction

A PA2227 deletion mutant was constructed by homologous recombination in strain PAO1 using PCR-overlap [26]. Two PCR fragments were amplified from PAO1 colony lysates using primer pairs vqsM-upR/vqsM-Bam and vqsM-dnF/vqsM-Hind. A 1.4 kbp fragment was obtained containing an internal deletion of 283 bp in the *vqsM* coding region. This fragment was purified and digested with *Bam*HI and *Hin*dIII restriction enzymes and ligated into the suicide vector pME3087 [27]. After triparental mating with the helper plasmid pRK2013 [28] and strain PAO1, dilutions were plated on LB-agar plates supplemented with tetracycline (Tc, 50 mg/l) and chloramphenicol (Cm, 10 mg/l, counter selection against *E. coli* donor strain). Individual colonies were streaked on LA-Tc plates and Tc-susceptible clones were enriched as described previously [27]. Three Tc-susceptible clones were tested by PCR amplification for loss of a 200 bp fragment in the *vqsM* gene, using primers vqsM-Bam and vqsM-Hind. All three clones were found to have the expected deletion. The PCR fragment was sequenced and shown to have a 283 bp deletion yielding strain PT1842.

### QS-related Phenotypes

LasB-elastase was measured in culture supernatants using the Elastin Congo Red (ECR) assay. Strains were grown overnight in 2 ml of LB and inoculated into fresh PB medium [29]. After 7 h growth (OD_600_ = 1.9–2.1), 0.5 ml of culture were centrifuged at 14,000 rpm for 5 min. Fifty µl of the supernatant were added to an ECR suspension (4 mg/ml) and incubated for 16 h at 37°C on a rotator. After pelleting the remaining ECR powder at 14,000 rpm for 5 min, the absorbance of the supernatant was measured at 495 nm in a spectrophotometer. Elastase activity was expressed as OD495/OD600. Pyocyanin was quantified in 0.75 ml of supernatant of cultures grown for 20 h in glycerol-alanine medium [30]. Absorption was measured at 690 nm and divided by the optical density of the cell pellet after suspension in 0.75 ml H_2_O (OD690/OD600). Rhamnolipid production was estimated on modified SW blue agar plates as described previously [22]. Virulence of strains was tested in a *Dictyostelium discoideum* host model as described previously [31], [32].

### Quantification of 3-oxo-C12-HSL

The *E. coli* strain JM109 harbouring the *lasR* gene and a *lasI*-*luxCDABE* fusion on plasmid pSB1075 [33] was used to determine 3-oxo-C12-HSL directly in culture supernatants. These were collected at regular time points during growth in LB medium. After centrifugation, supernatants were filtered (22 µM Millipore filters) and filtrates frozen immediately at −20°C. The *E. coli* bioassay strain was grown overnight in LB and diluted 1∶10 into fresh LB medium. 190 µl aliquots of this suspension were seeded in a 96 well microtiter plate in triplicates. 10 µl of thawed culture filtrate were added per well. A 1∶10 dilution series of synthetic 3-oxo-C12 HSL was included to calculate the autoinducer concentration in the supernatants. The plate was incubated at 30°C with intermittent shaking in a microplate reader (Synergy H1, BioTek) and the luminescence and absorption at 600 nm were monitored at 30 min intervals. Luminescence reached a plateau after 2.5 h of incubation. These values were used for the final calculation of 3-oxo-C12 HSL concentrations.

### Intracellular Detection of Externally Added Autoinducers

Exogenously added 3-oxo-C12-HSL and PQS were detected intracellularly upon induction of the *lasI-gfp* (pLIGF1) and the *pqsA-gfp* (ppqsA1) fusion vectors. 0.5 ml of an LB overnight culture of strains carrying these fusions were centrifuged, washed and suspended in the same volume of PB. Ten ml of the cell suspension were seeded in microtiter plates containing 190 ml of PB medium containing 100 µg/ml carbenicillin and supplemented or not with 5 µM 3-oxo-C12-HSL or 50 µM PQS (final concentrations). Plates were incubated in a BioTek Synergy H1 plate reader with intermittent shaking for 24 h at 37°C. Absorption at 600 nm and fluorescence (excitation 485 nm-emission 528 nm) were monitored at 30 min intervals.

### Induction of the Type Three Secretion System (TTSS)

Strains were grown overnight in LB and inoculated 1∶10 into fresh LB medium supplemented or not with 5 mM EGTA and 20 mM MgCl_2_. After incubation for 4 h at 37°C with agitation, 1.5 ml of culture was centrifuged for 10 min at 13,000 rpm. Proteins in the supernatant were precipitated with 13% tricholoracetic acid (final concentration) for 30 min on ice. The precipitate was recovered by centrifugation for 30 min at 13,000 rpm and the pellet was washed twice in cold acetone. The final pellet was suspended in 50 µl 1 × SDS sample loading buffer. Fifteen µl were run on a 10% acrylamide (acryl-bisacrylamide 37∶0.5) SDS PAGE minigel. The gel was stained with 0.1% Coomassie blue solution.

### Gene Expression Analysis

RNA was isolated during exponential growth (OD600 = 2.0–2.2) in LB medium. 0.25 ml aliquots were removed and treated with RNA protect (Qiagen). RNA was extracted using RNeasy spin columns (Qiagen). Total RNA was treated with RQ1 DNAse (Promega). cDNA was obtained after reverse transcription using random hexamer primers and ImPromII reverse transcriptase (Promega). The amount of cDNA was determined by qRT-PCR (Quantitect Sybr Mix, Qiagen) in a RotorGene 3000 (Corbette Research) RealTime PCR machine [34]. All measurements were standardized using the *rpsL* gene and values are given as ratios *geneX*/*rpsL* compared to the PAO1 wild strain.

## Results

### Selection and Characterization of QS-deficient Clones

**Figure 1:**
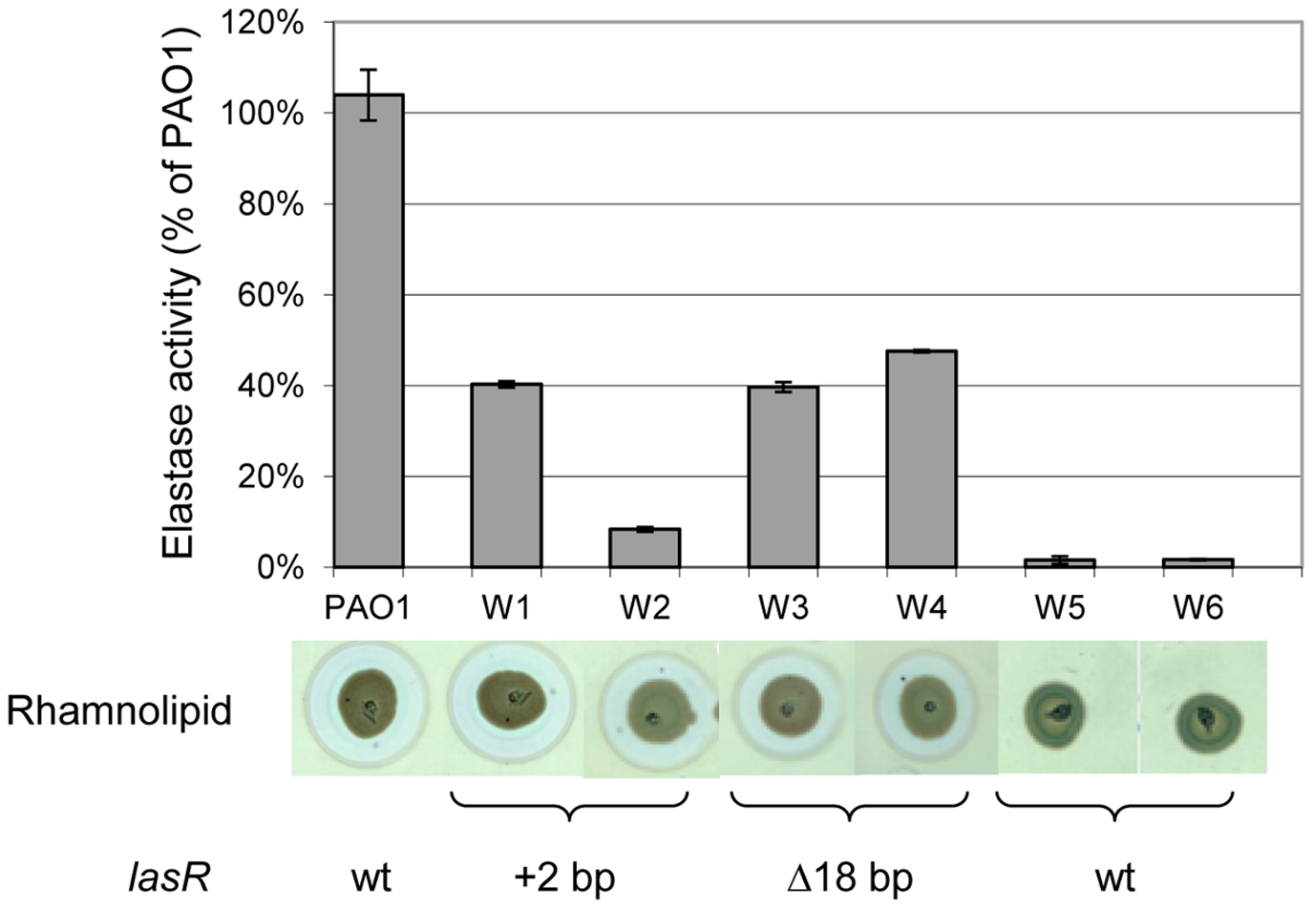
Quorum-sensing dependent virulence factor production by wild type PAO1 and selected QS-deficient clones. Elastase production was determined using the Elastin-Congo Red (ECR) assay at three different occasions. Rhamnolipid production was estimated after 48 h growth on modified SW-blue plates [22]. The mutations in the *lasR*-coding region identified in the six isolates are indicated below. Values are the average of triplicate determinations and error bars indicate standard deviations.

**Figure 2:**
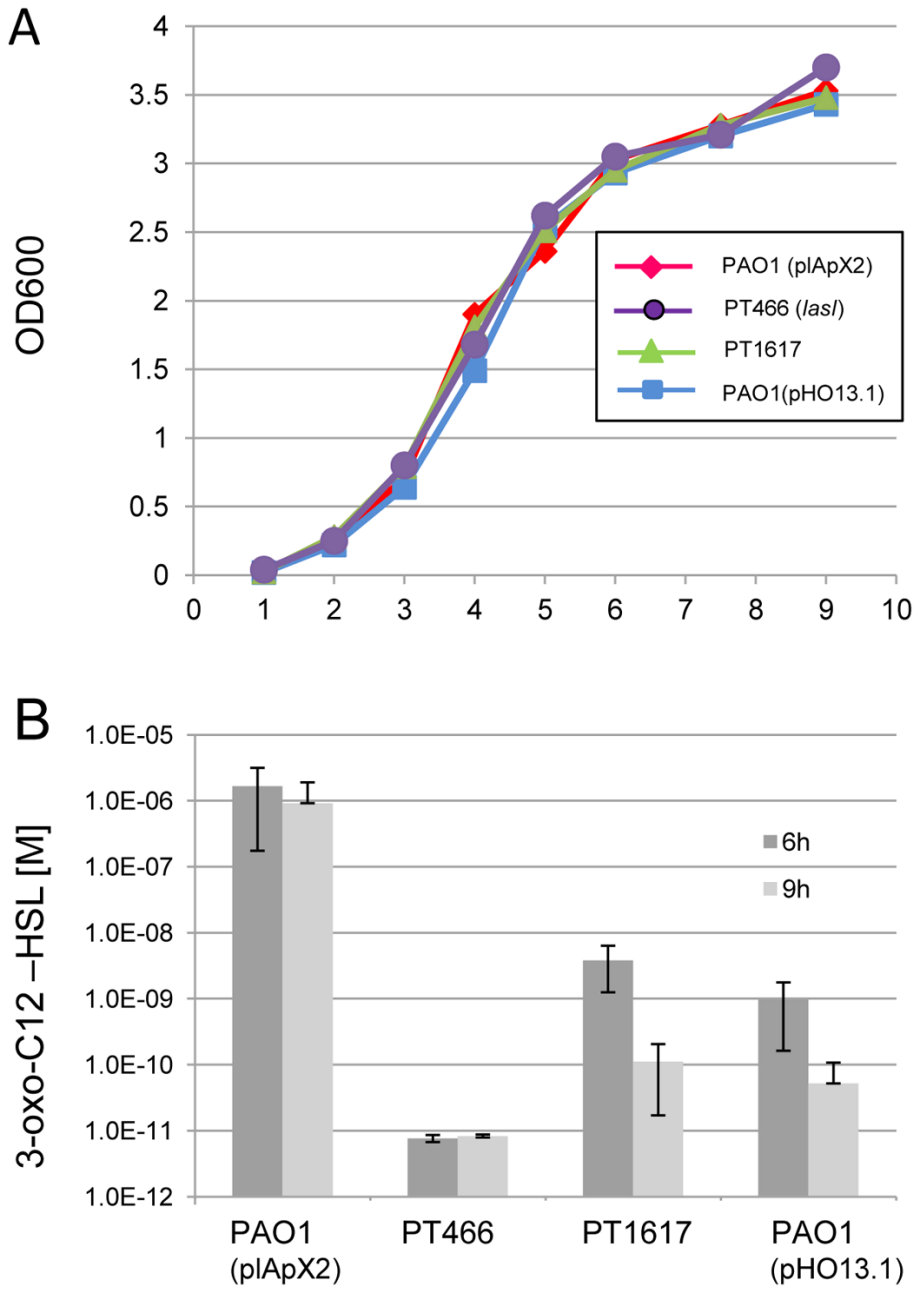
Growth curves and 3-oxo-C12-HSL autoinducer concentrations in culture supernatants. *P. aeruginosa* strain PAO1 carrying the empty vector pIApX2, a *lasI* mutant of PAO1 (PT466), the QS-deficient deletion mutant (PT1617) and PAO1 harbouring plasmid pOH13.1, expressing PA2226 showed comparable growth curves in LB medium at 37°C (A). Culture supernatant samples were taken at indicated time points and 3-oxo-C12-HSL concentrations were determined using the *E. coli* reporter system based on a *lasR*-*lasI::luxCDABE* fusion harboured on plasmid pSB1075 [33] (B). Results for 3-oxo-C12-HSL measurements are shown as average values and standard deviations from two independent experiments performed in triplicate.

Based on previous results with *lasR* mutants [8], [21], we devised a simple genetic screen to detect spontaneous QS-deficient clones during growth under QS-requiring conditions. To this end we introduced a *lasB*-*lacZ* transcriptional fusion carried on plasmid pTS400 [35] into PAO1. Transformants were grown in buffered PB medium under static conditions in microtiter plates. The Bacto^TM^Peptone (Difco) used contains 15% of readily metabolized low MW (<250 dal) products, while the remainder are high MW digests (Difco product information sheet) requiring further enzymatic degradation by secreted proteases. After 72 h, dilutions were spread on LB-agar plates supplemented with carbenicillin and X-Gal. After incubation for 24 h at 37°C, white colonies appeared at a frequency of approximately 1% among the light blue colonies. These QS-deficient clones could potentially be QS-cheaters, which were selected once the media was depleted for readily metabolizable C-sources and requiring QS-dependent secreted proteases for subsequent growth. Six white colonies (W1–W6) were selected for further analysis. We first tested production of the classical QS-dependent virulence factors elastase and rhamnolipids. All six clones showed reduced elastase production (10–20% of PAO1 wild type level). While clones W1 to W4 still produced wild-type levels of rhamnolipids, these were undetectable in clones W5 and W6 (Figure 1). Similarly, production of pyocyanin was undetectable in strains W5 and W6 (data not shown). To identify the genetic alteration responsible for reduced QS expression, we first sequenced the *lasR* gene in all six clones. As expected, clones W1 to W4 carried mutations in the *lasR* coding sequence. Clones W1 and W2 had acquired an additional CG bp at position 415 of *lasR*, while clones W3 and W4 harboured an 18 bp deletion starting 2 nt upstream of the ATG initiation codon of *lasR*. Interestingly, clones W5 and W6 carried a wild type *lasR* allele, and we therefore sequenced their QS-regulator genes *rhlR*, *pqsR*, *lasI* and *rhlI*. None of these regulators was mutated in the two clones. We focused on clone W5, which showed the same QS-phenotype as W6, for further analysis and selected a derivate, which had spontaneously lost the *lasB*-*lacZ* fusion plasmid pTS400. This isolate was named PT1617, it exhibited the same QS-profile as the original W5 clone (see below and data not shown), and was used for all further analyses. As expected for a QS-cheater, PT1617 grew slower than PAO1 in buffered PB medium used during the selection, but had comparable growth in a casamino acid (CAA) medium (Figure S1). To further characterize this mutant, we measured the production of the autoinducer molecule 3-oxo-C12-HSL during growth in LB-medium. The *lasI* mutant PT466 [22], was used as the negative control (background level). Strains PAO1, PT466 and PT1617 showed comparable growth (Figure 2A). In PAO1, 3-oxo-C12-HSL reached a plateau with a maximal concentration of 1 µM. In contrast, 3-oxo-C12-HSL levels in strain PT1617 reached a concentration of 1 nM, which then decreased at 9 h. We also measured AHQs, which are controlled by the MvfR (PqsR) QS-system, in culture supernatants using the PAO1*pqsA*::*lux* reporter strain [36]. While AHQs were present in PAO1 and an *rhlR* mutant, they were undetectable in supernatants of a *lasR* mutant and strain PT1617 (Figure S2A). We further tested the virulence of both strains in a *Dictyostelium discoideum* host model, affected by the *rhl* system [31]. Ten *D. discoideum* cells could grow on a lawn of PT1617 bacteria, while >10,000 amoebal cells were unable to grow on a lawn of PAO1 (Figure S2B), reflecting a dramatic reduction in virulence for mutant PT1617. In summary, our data show that mutant PT1617 is severely affected in the induction of the three QS-signalling systems, highlighted by decreased expression of QS-dependent virulence factors and a reduced virulence, despite the presence of intact QS-regulator genes.

### Whole Genome Sequencing of Strain PT1617

**Figure 3:**
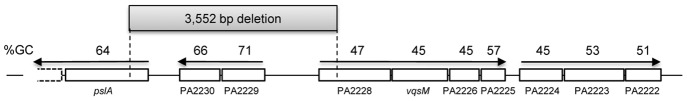
DNA region of PAO1 surrounding the 3,552 *PslA* is the first gene of the *psl* polysaccharide synthesis operon. Arrows indicate putative operon structures. The numbers above the gene symbols indicate the GC content (%GC). The average GC content of the *P. aeruginosa* genome is 66%.

To unravel the genetic alteration(s) responsible for the observed QS-phenotype, we sequenced the entire genome of strain PT1617. Two alterations were identified and confirmed by PCR amplification and sequencing. The first alteration was a G to A transition in ORF PA0439 coding for an oxydoreductase and resulting in an Ala to Thr substitution at codon 29. However, complementation with the cloned wild-type allele did not restore the QS-deficiency of strain PT1617 (data not shown). The second alteration was a deletion of 3,552 bp (position 2,450,516–2,453,867 in the PAO1 genome) encompassing four ORFs: PA2228, PA2229, PA2230 and *pslA*. While ORFs PA2229 and PA2230 were completely absent in mutant PT1617, only the 5′ coding regions of PA2228 and *pslA* were deleted (Figure 3). *PslA* is the first gene in the exopolysaccharide synthesis operon involved in biofilm formation in *P. aeruginosa* [37]. PA2229 and PA2230 code for two hypothetical proteins conserved in bacteria. PA2228 is the first gene of an operon containing the previously characterized transcriptional regulator gene *vqsM*. A Tn5 mutant in *vqsM* was reported to be QS-deficient and less virulent in a *Caenorhabditis elegans* model [19].

We next verified whether one of the deleted genes was responsible for the observed QS-deficiency. ORFs PA2229 and PA2230 were cloned together in the expression vector pIApX2 and the resulting plasmid pHO3.1 introduced into strain PT1617, but QS deficiency could not be restored. Similarly, expression of ORF PA2228, which is partially deleted in mutant PT1617, from plasmid pHO1.1 did not restore QS-proficiency (data not shown), suggesting that the QS-deficient phenotype of PT1617 is not due to the deletion of one of these three ORFs.

### Expression of QS and *vqsM*-operon Genes

**Figure 4:**
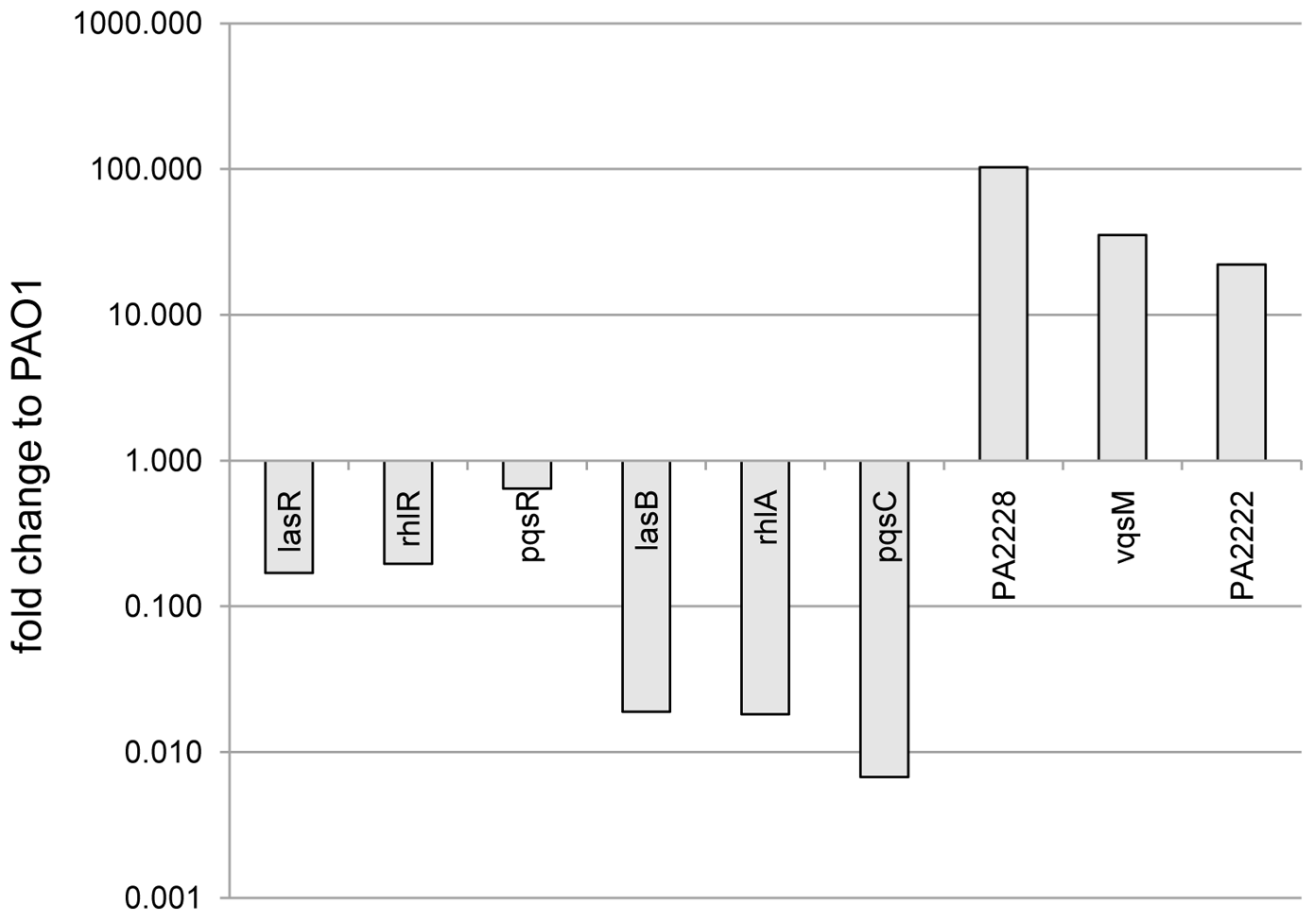
Gene expression determined by qRT-PCR in strain PT1617. Expression of QS-regulator (*lasR*, *rhlR*, *pqsR*), QS-target (*lasB*, *rhlA*, *pqsC*) and *vqsM*-operon genes (PA2228, *vqsM*, PA2222). Values show a representative experiment from three independent qRT-PCR determinations. RNA was extracted from exponentially growing cells (OD_600_ = 2) in LB medium. cDNA and qRT-PCR analysis was performed as described in Materials and Methods. Gene expression was normalized to the expression of the *rpsL* housekeeping gene and compared to the levels measured in strain PAO1.

To verify whether the absence of QS-dependent virulence factors in strain PT1617 was reflected in reduced QS-gene expression, we measured by qRT-PCR the expression of QS-target (*lasB*, *rhlA*, *pqsC*) and QS-regulator genes (*lasR*, *rhlR*, *pqsR*), as well as of three genes from the *vqsM* operon (PA2228, *vqsM*, PA2222) (Figure 4). As expected, expression of QS-regulator genes (*lasR*, *rhlR*, *qscR*) was reduced between 3 to 9 fold, while expression of target genes (*lasB*, *rhlA, pqsC)* was reduced approximately 100-fold in mutant PT1617 compared to PAO1.

**Figure 5:**
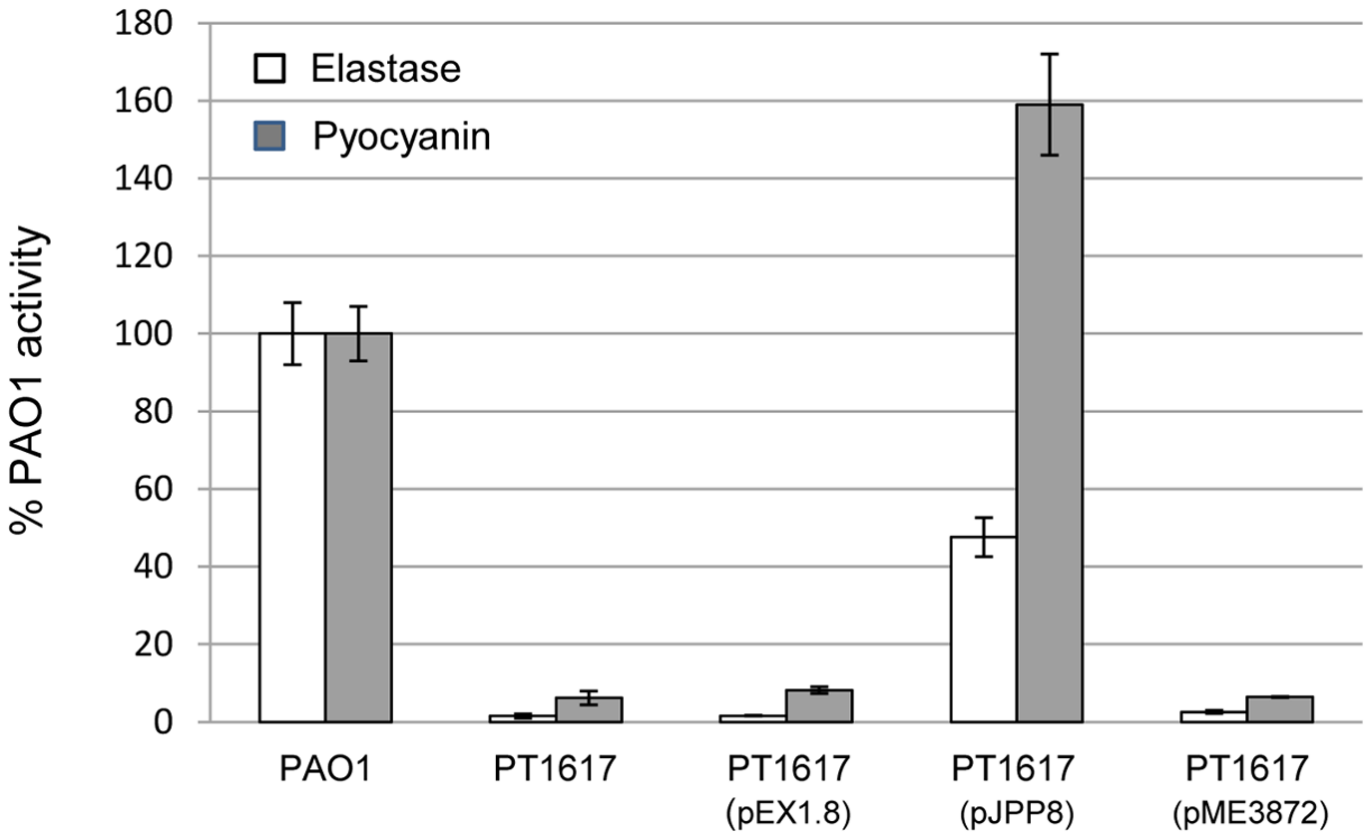
Complementation of QS-deficient mutant PT1617 with QS-regulator genes. Elastase and pyocyanin production was determined in culture supernatants. For elastase activity, strains were grown in PB-medium for 7 h, while pyocyanin was determined after 22 h of growth in glycerol-alanine medium. *lasR* was expressed from its own promoter on pME3872. Expression of *rhlR* from plasmid pJPP8 was induced by addition of 2 mM IPTG. Vector control data are shown with pEX1.8. Values show the average and standard deviation from triplicate cultures.

We further tempted to complement PT1617 with the QS-regulator genes *lasR* and *rhlR*. LasR, expressed as a single copy from its own promoter did not rescue any of the QS phenotypes tested, (Figure 5), even when *lasI* was co-expressed on a plasmid from a constitutive promoter (data not shown). However when *rhlR* was expressed from an inducible ptac promoter on plasmid pJPP8, we observed partial restoration of elastase activity and high production of pyocyanin (Figure 5). To test whether QS-molecules could be provided exogenously and perceived by the intracellular reporter fusion plasmids pLIGF1 (*lasI*-*gfp*) and ppqsA1 (*pqsA*-*gfp*), we measured fluorescence expression in PAO1 and PT1617 in the presence and absence of 5 µM 3-oxo-C12-HSL or 50 µM PQS, respectively. While the *lasI* mutant PT466 and the *pqsA* mutant of PAO1 responded to the addition of their cognate signal added to the culture medium, PT1617 showed no increase in *gfp* expression (Figure S3). This suggests that these signalling molecules might not be taken up or perceived intracellularly in the mutant.

**Figure 6:**
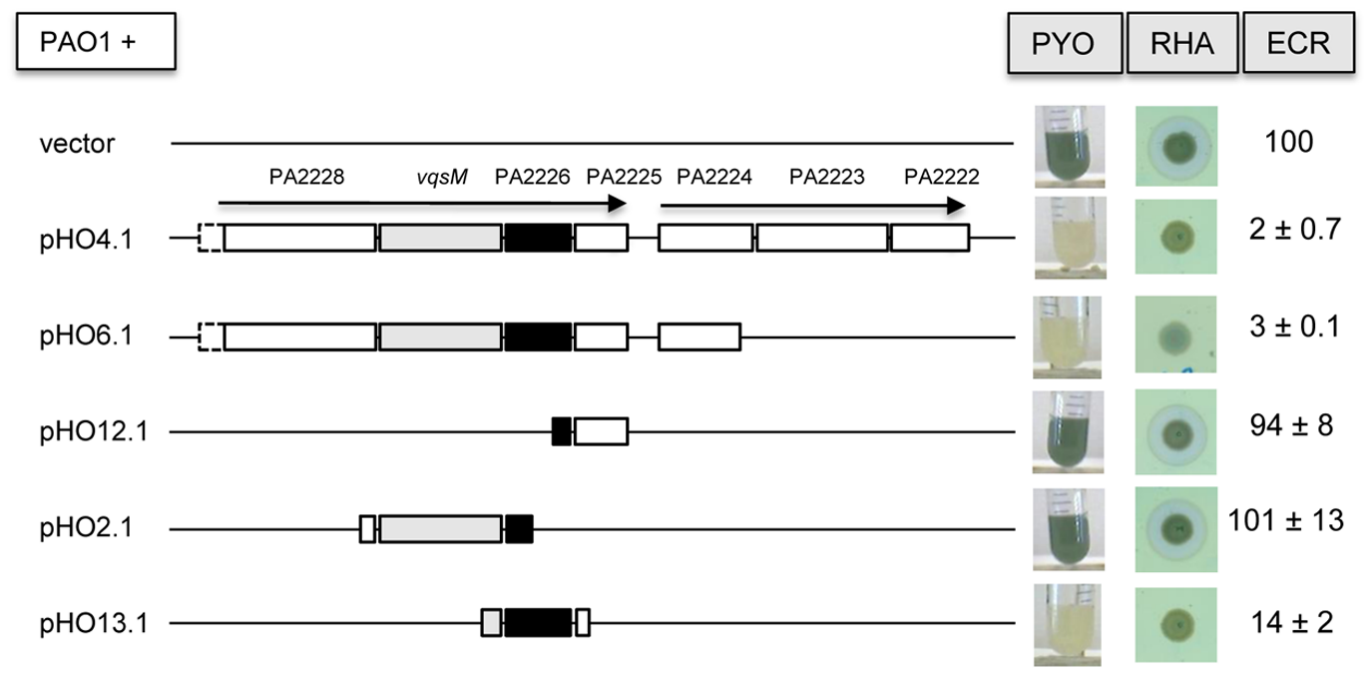
Expression of genes from the *vqsM* DNA region and their effect on pyocyanin production in PAO1. The indicated DNA region was amplified by PCR from strain PAO1 and cloned into the expression vector pIApX2. Pyocyanin, rhamnolipid and elastase production were measured as described in Material and Methods. Expression of PA2226 (black box) was sufficient to repress QS-dependent expression of pyocyanin, rhamnolipids and elastase. Elastase values are expressed as percentage of PAO1 carrying the vector pIApX2.

Unexpectedly, we observed an overexpression (20–100 fold compared to PAO1) of three genes belonging to the *vqsM* operon (PA2228, *vqsM*, PA2222) (Figure 4). We postulated that overexpression of one or several of these genes might be responsible for the QS-deficient phenotype of PT1617. We thus cloned a 6.0 kbp fragment of the *vqsM* operon, starting at the end of the deletion in ORF PA2228 in PT1617 (Figure 3) and extending until the end of PA2222, into the expression vector pIApX2 to obtain plasmid pHO4.1 (Figure 6). When pHO4.1 was introduced into PAO1, all three QS-dependent virulence factors (pyocyanin, rhamnolipids, elastase) were reduced to the same low levels as those observed in the deletion mutant PT1617 (Figure 6). Deletion of ORF PA2222, PA2223 and partially PA2224 from pHO4.1, yielding plasmid pHO6.1, still suppressed production of these virulence factors. This suggested that one or several of the remaining three genes on this plasmid, *vqsM*, PA2226 and PA2225, was responsible for QS-suppression. Individual subcloning of these three genes showed that overexpression of PA2226 alone (pHO13.1) was sufficient to confer a QS-deficient phenotype on PAO1. Moreover, expression of pHO13.1 in PAO1 reduced the amount of 3-oxo-C12-HSL in culture supernatants to similar concentrations as to the deletion mutant PT1617 (Figure 2). These data strongly suggest that overexpression of PA2226 is necessary and sufficient to abrogate expression of the three QS-systems in PAO1.

**Figure 7:**
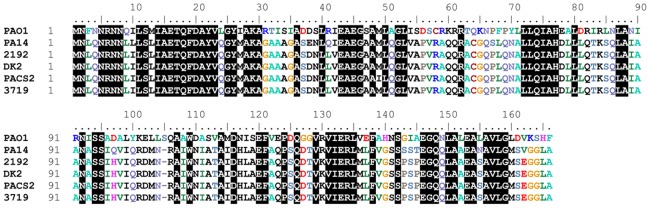
Sequence alignment of PA2226 from PAO1 and five ORFs from completely sequenced *P. aeruginosa* strains. PA2226 has 46% amino acid identity with an ORF (RL113) from PA14, located on pathogenicity island PAG1. This ORF is not preceded by a *vqsM* homologue in PA14. Identical residues are boxed in black.

BLAST searches for proteins homologous to PA2226 revealed an ORF in PA14 located on the pathogenicity island PAGI-1 (designated as RL113) [38], which shows 46% amino acid identity with PA2226 (Figure 7). Proteins identical to RL113 are also present in the sequenced *P. aeruginosa* strains PAC2, C3719 and 2192 (Figure 7). The function of these ORFs is not known. Search for protein motifs using PROSITE (ExPASy) did not yield any significant hits.

**Figure 8:**
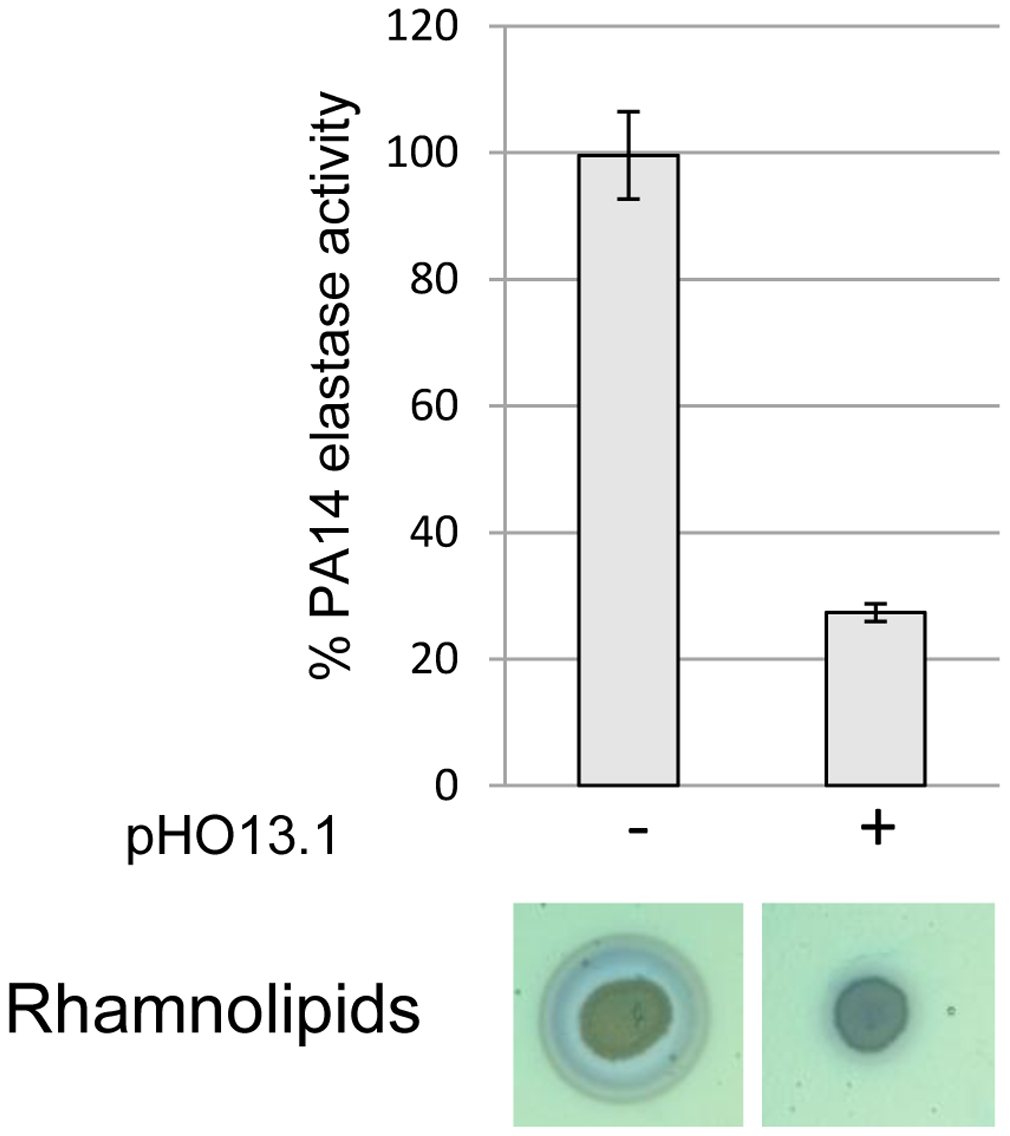
Effect of PA2226 expression in PA14. Expression of PA2226 from plasmid pHO13.1 diminished expression of the QS-dependent virulence factors rhamnolipids and elastase, despite the absence of the *vqsM* gene in PA14, suggesting a VqsM-independent effect on QS-expression.

The *vqsM* operon is located on a transposable element and the question arises, whether overexpression of PA2226 also affects QS-expression in strains that do not carry this operon, as for instance PA14. We therefore introduced plasmid pHO13.1 into PA14 and assessed production of elastase and rhamnolipids. Production of both QS-dependent virulence factors was drastically reduced in the presence of pHO13, but not with the pIApX2 vector alone (Figure 8), suggesting that PA2226 acts on QS-gene expression independently of the genetic background.

### PA2226 and PA2225 Affect TTSS Expression

**Figure 9:**
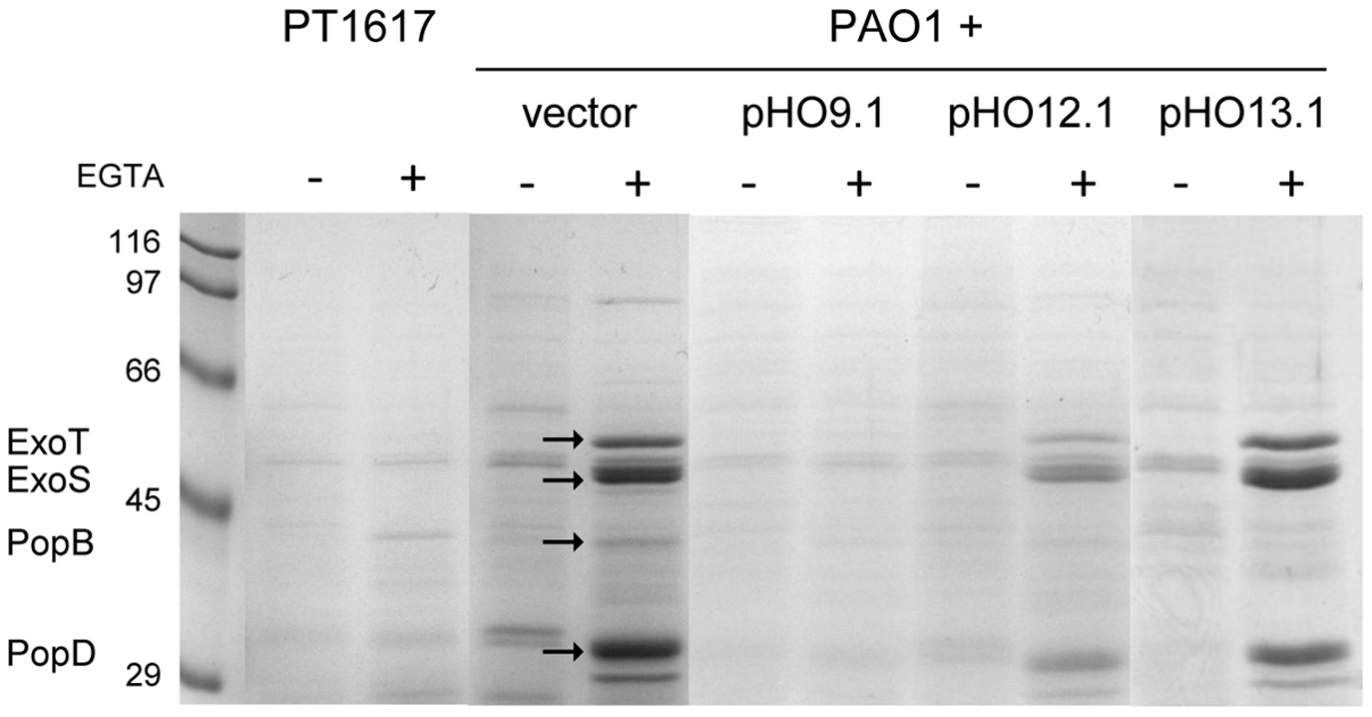
Effect of PA2226 on induction of TTSS in PAO1. PAO1 cells harbouring vector pHO9.1 (PA2226 and PA2225), pHO12.1 (PA2225) and pHO13.1 (PA2226) were grown in LB-medium for 4 h, supplemented or not with 5 mM EGTA, and 20 mM MgCl_2_. Proteins in the supernatants were precipitated with TCA. Samples were run on 10% SDS-PAGE and stained with Coomassie. Secreted proteins of the TTSS are indicated on the left and marked by arrows. Expression of PA2226 and PA2225 was sufficient to prevent induction of the TTSS.

Microarray analysis in the *vqsM* transposon mutant has shown decreased expression of the *pscQ* gene, coding for a translocation protein of the TTSS apparatus [19]. We therefore tested the induction of the TTSS in mutant PT1617. Expression of the TTSS is induced by Ca^2+^ depletion upon addition of EGTA. While PAO1 culture supernatants showed the presence of at least four proteins with MWs compatible with the secreted TTSS proteins ExoS (48 kDa), ExoT (48 kDa), PopB (40 kDa) and PopD (31 kDa), these proteins were absent from supernatants of mutant PT1617 in both induced and uninduced conditions (Figure 9). To determine which of the *vqsM* operon genes overexpressed in PAO1 was responsible for the prevention of TTSS-induction, we repeated these experiments with various plasmid constructs (Figure 9). The data indicate that unlike for QS-repression, which requires only PA2226 (pHO13.1), prevention of TTSS-induction requires both PA2226 and PA2225 (pHO9.1), suggesting a concerted action of both proteins on the expression of the TTSS.

### A *vqsM* Deletion does not Affect QS-regulation

**Figure 10:**
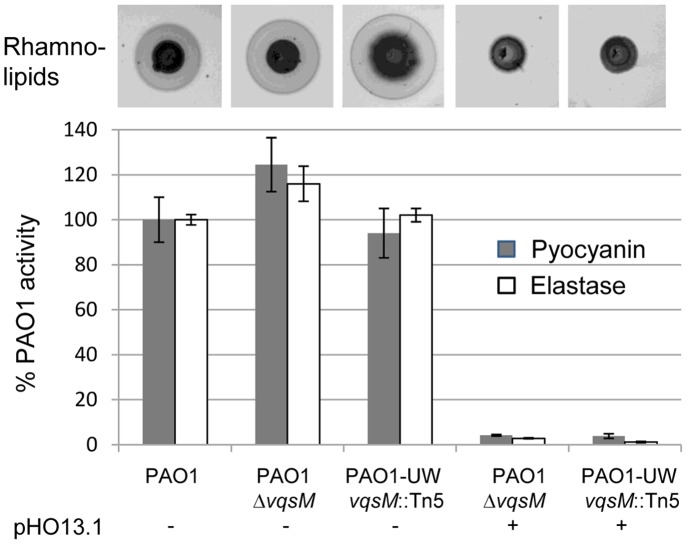
QS-dependent virulence factor production by *vqsM* mutants. Pyocyanin, rhamnolipid and elastase production were determined as described in Material and Methods.The defined *vqsM* deletion mutant of PAO1 (PT1842), and the *vqsM* mutant (PAO1-UW) of the PAO1 transposon library [39], were not affected in QS-dependent virulence factor expression. When PA2226 was expressed from plasmid pOH13.1, both *vqsM* mutants showed the same QS-negative phenotype, suggesting that VqsM is not involved in QS-regulation.

The QS-phenotype of mutant PT1617, which is due to overexpression of PA2226, was very similar to the phenotype of the *vqsM* transposon mutant reported by Dong et al. [19]. We therefore constructed a *vqsM* deletion mutant of PAO1 by homologous recombination in our PAO1 strain background (PT1843), and ordered the *vqsM*-*Tn*5 mutant from the PAO1 transposon mutant library [39]. Both *vqsM* mutants were unaffected in the expression of elastase, rhamnolipids and pyocyanin (Figure 10), suggesting that VqsM is not involved in QS-regulation. When we expressed PA2226 from plasmid pHO13.1 in the *vqsM* deletion mutant and in the *vqsM* transposon mutant, QS-dependent factors were reduced to the same low levels as in PAO1 carrying pHO13.1. These data demonstrate that the effect exerted by PA2226 does not require the presence of the VqsM transcriptional regulator and that VqsM is unlikely to be involved in QS-regulation.

## Discussion

In the present study, we have devised a genetic screen for the detection of QS-deficient clones emerging under QS-requiring growth conditions in strain PAO1. We have used the *lasB* promoter as a general QS-output signal, since under standard growth conditions (LB) its expression is regulated by the *las*, *rhl* and *pqs* QS-systems [40]. Besides the expected mutants in the *lasR* gene, we also identified two mutants that did not show alterations in any of the three QS-transcriptional regulators. Wilder et al. recently showed that cheaters emerged following spontaneous mutations in *lasR* and *pqsR* genes, but not in *rhlR* after 12 days of continuous sub-culturing [21]. Under our static growth conditions in microtiter plates for 72 h without sub-culturing, we obtained white colonies (representing QS-deficient clones, cheaters) at a frequency close to 1%. This indicates that QS-cheaters had probably emerged early and were invading the wild-type population at the time of sampling. This is highlighted by the fact that among the six white colonies analysed we found two identical clones for each of the two different *lasR* alleles and the deletion mutant. Genome sequencing revealed that both *lasR* wild type clones harbour an identical deletion of 3,552 bp located next to the previously described QS-regulator gene *vqsM*. This deletion resulted in a dramatic reduction in expression of all three QS-systems including both regulatory and target genes. Down-regulation of the *las* system despite a wild type LasR protein explains reduced expression of the *lasB*-*lacZ* reporter in the initially selected strain W5. The phenotype of deletion mutant PT1617 was strikingly similar to the one of the *vqsM* transposon mutant, described by Dong et al. [19]. However, in strain PT1617 *vqsM* was overexpressed. Surprisingly, according to transcriptomic data presented in Table 2 of this article, the genes located downstream of *vqsM* (PA2226 to PA2222) were also overexpressed (between 22- to 120–fold) in their *vqsM* transposon mutant. The reason for this overexpression is not clear. One possibility could be the presence of a promoter within the *pslA* gene but in opposite orientation, which would drive expression of genes PA2228 to PA2222. Alternatively, an operator region upstream of PA2228, recruiting a putative repressor of the *vqsM* region, could have been removed leading to constitutive expression of ORF PA2228 and downstream genes. However analysis using the BPROM program (www.softberry.com) did not reveal any significant prokaryotic promoter sequences in PA2228.

The expression levels of genes PA2227 to PA2222 observed in PT1617 are comparable to those reported for the *vqsM* mutant [19]. In light of our results, it seems likely that overexpression of the genes located downstream of *vqsM* were responsible for the lack of QS-gene expression, rather than the mutation in *vqsM* itself. Indeed, the defined *vqsM* deletion mutant and the *vqsM* transposon mutant from the PAO1 library did not differ from PAO1 in the expression of QS-dependent virulence factors. In both mutants, overexpression of PA2226 resulted in down-regulation of QS-dependent virulence factors as observed in PAO1. Taken together, these data demonstrate that *vqsM* is not involved in QS-regulation.

Plasmid-mediated expression of the genes downstream of PA2228 showed that expression of *qsrO* (PA2226) alone was necessary and sufficient to abolish all three QS circuits in PAO1 as well as in PA14. Co-expression of PA2225 further prevented induction of the TTSS. The TTSS expression was previously shown to be negatively regulated by the *rhl* QS-system during *in vitro* culture [41]. Mutant PT1617 however was deficient in both QS and TTSS induction, suggesting that both systems are independently affected in this mutant. The function of these putative proteins is unknown and no homologues or conserved domains were found by BLAST searches in the NCBI database. PSORT identified a predicted typeII (lipoprotein) export signal sequence in ORF PA2225 (www.pseudomonas.com). Complementation with QS-regulator genes showed partial (elastase) and complete restoration (pyocyanin) by plasmid mediated expression of *rhlR*, while *lasR* and *lasI* expression had no effect. Dong et al. reported that *lasR* and *pqsR* expression were unable to restore QS proficiency in their *vqsM* transposon mutant, however *rhlR* complementation was not tested [42].

Intriguingly, while RhlR recognizes and binds the diffusible C4-HSL signalling molecule, LasR and PqsR are activated upon binding of the hydrophobic molecules 3-oxo-C12-HSL and PQS. Intracellular 3-oxo-C12-HSL concentrations are influenced by the level of expression of the MexAB-OprM efflux pump [43], while HHQ, a PQS precursor is transported by the MexEF-OprN efflux pump [44]. Both molecules may also be incorporated into membrane vesicles [45]. Whether these mechanisms are influenced by the overexpression of QsrO remains to be determined.

For the moment, we do not know how overexpression of QsrO (QS-repressing ORF) affects the QS-systems. It is unlikely to result from a hierarchical effect, since a single knock out of any of the three QS-regulators does not lead to the same dramatic shut down of QS as in mutant PT1617. Since we could not complement the QS-deficient phenotype of mutant PT1617 by exogenous addition of 3-oxo-C12-HSL or PQS, it is conceivable that hydrophobic autoinducers (3-oxo-C12-HSL and PQS) cannot penetrate into the cells [19]. This could result from modification of membrane permeability or activation of efflux mechanisms. However, we cannot exclude that the level of all three QS-regulators in PT1617 is too low to perceive any QS-signal that would diffuse into the cell.

Another possibility could be reduced autoinducer synthesis. All three QS-signals contain a fatty acyl side chain, whose synthesis involves the FabY β-ketoacyl synthase (PA5174) [46]. Interestingly Dong et al. found in the transcriptome analysis of their *vqsM* mutant, which also overexpresses genes downstream of *vqsM*, a 2-fold reduction of *fabY* expressions and a 60-fold reduced expression in *fabH2* (PA3333), another β-ketoacyl synthase. A knockout mutant of PA5174 showed indeed markedly reduced production of QS-dependent virulence factors and autoinducers [46].

If such a mechanism could be mimicked by drugs it would be an ideal target for anti-virulence strategies since it abolishes in a single process all three known QS systems in *P. aeruginosa*.

## Supporting Information

Figure S1 **Growth of PAO1 and PT1617 in PB and casamino acids (CAA) medium.** Overnight cultures grown in 2 ml LB medium were diluted 1∶50 into 200 ml of PB medium buffered with 1×M9 salts medium without NH_4_Cl or 1×M9 salts without NH_4_Cl medium supplemented with 1 mM MgSO_4_ and 0.5% CAA as C- and N-source. Growth (OD600) was measured at 37°C with intermittent shaking in a BioTek Synergy H1 plate reader. As expected for a cheater, growth of PT1617 was reduced in the complex PB medium, but was comparable to PAO1 when the readily available CAAs were provided as a C-source. Data represent average values of triplicate wells. Standard deviations represented less than 10% of the average value and were omitted for clarity.
(TIF)

Figure S2 **AHQ production in culture supernatants and virulence assay in** ***Dictyostelium discoideum*** **.** AHQs were extracted with acidified ethyl acetate from supernatants of cultures grown for 18 h in LB-medium. After evaporation of the solvent, the residue was resuspended in 10 ml methanol. Two ml samples were spotted on silica 60_F254_ TLC plates and developed in a dichloromethane:methanol (95∶5) mixture. Plates were dried and spots visualized under UV-light. PQS and HHQ were undetectable in both the *lasR* mutant of PAO1 and PT1617 (A). Indicated amounts of *D. discoideum* cells were deposited on a lawn of *P. aeruginosa* and incubated at 25°C. The appearance of plaques resulting from growth of the amoeba on bacteria was scored after 10 days. While PAO1 inhibited growth of the amoeba, the *lasR* mutant and PT1617 were both completely avirulent and permitted growth of amoebal cells (B).
(TIF)

Figure S3 **Intracellular detection of externally added 3-oxo-C12-HSL and PQS.** 3-oxo-C12-HSL was detected using the *lasI-gfp* fusion vector pLIGF1 harbored by strains PAO1, a *lasI* mutant (PT466) and PT1617. PQS was detected using the *pqsA-gfp* fusion vector ppqsA1 carried by strains PAO1, a *pqsA* mutant (PT1833) and PT1617. Strains were grown in PB medium supplemented (+) or not with 5 mM 3-oxo-C12-HSL or 50 mM PQS (final concentration) in microtiter plates (200 ml/well). Absorption at 600 nm (A,C) and fluorescence (RFU) (B,D) were monitored for 24 h. Data represent average values of triplicate wells. Standard deviations represented less than 10% of the average value and were omitted for clarity.
(TIF)
