# Supplementary figures and images for: QsrO a Novel Regulator of Quorum-Sensing and Virulence in Pseudomonas aeruginosa
Source: PLoS One. 2014 Feb 13;9(2):e87814. doi: 10.1371/journal.pone.0087814 (PMC3923755; doi:10.1371/journal.pone.0087814)

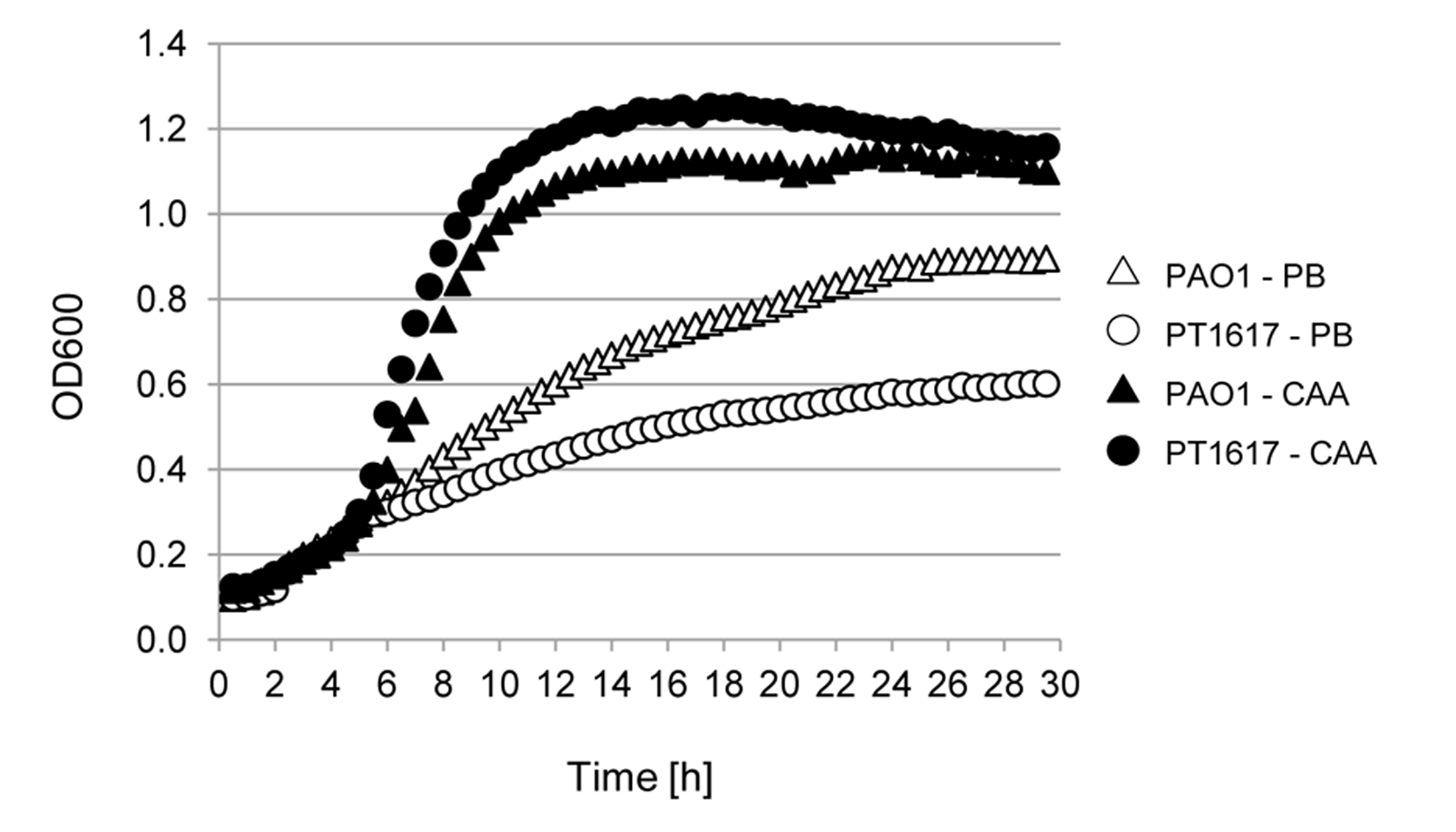

Supplement: Figure S1 — Growth of PAO1 and PT1617 in PB and casamino acids (CAA) medium. Overnight cultures grown in 2 ml LB medium were diluted 1∶50 into 200 ml of PB medium buffered with 1×M9 salts medium without NH4Cl or 1×M9 salts without NH4Cl medium supplemented with 1 mM MgSO4 and 0.5% CAA as C- and N-source. Growth (OD600) was measured at 37°C with intermittent shaking in a BioTek Synergy H1 plate reader. As expected for a cheater, growth of PT1617 was reduced in the complex PB medium, but was comparable to PAO1 when the readily available CAAs were provided as a C-source. Data represent average values of triplicate wells. Standard deviations represented less than 10% of the average value and were omitted for clarity. (TIF) [file pone.0087814.s001.tif]

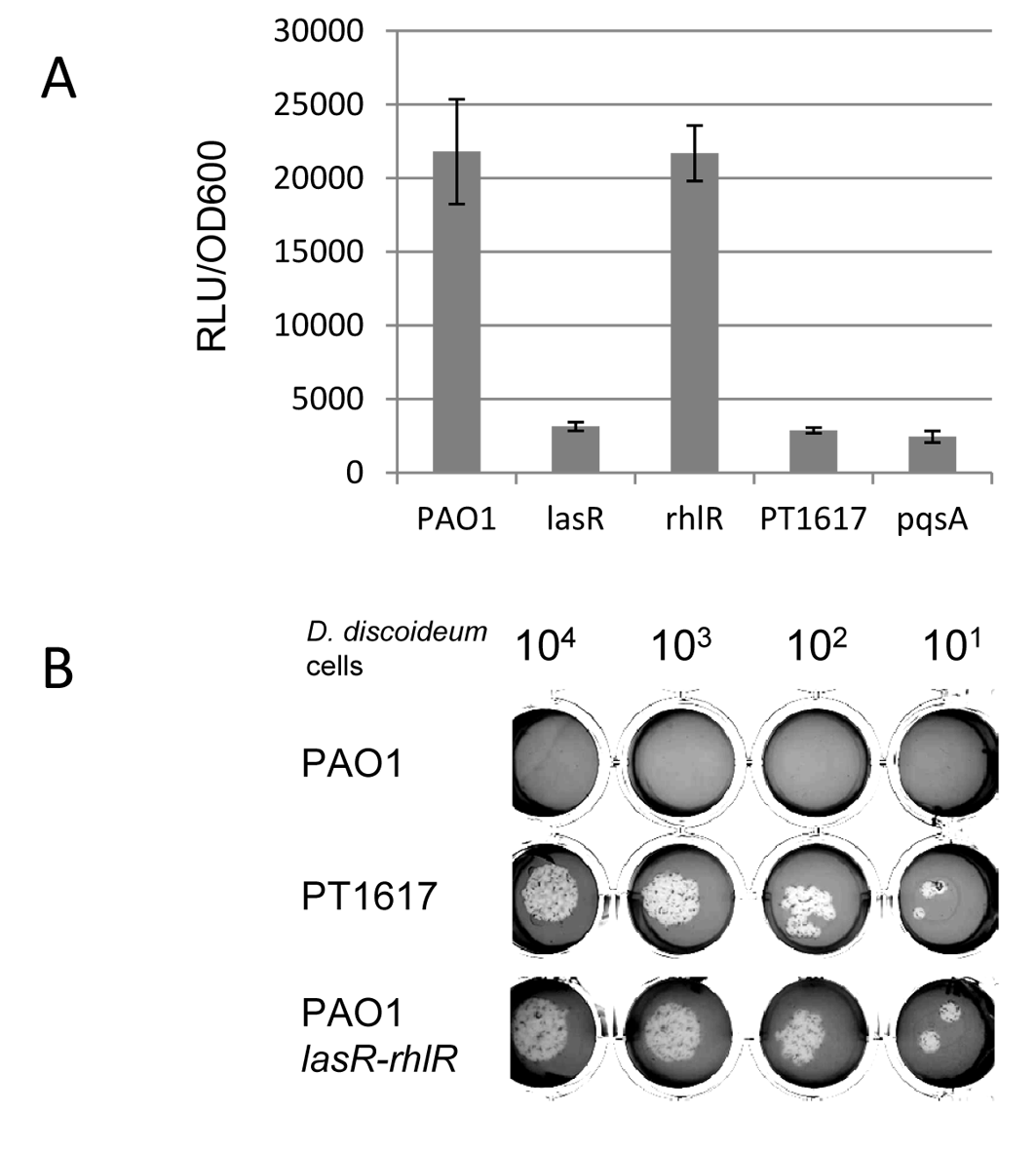

Supplement: Figure S2 — AHQ production in culture supernatants and virulence assay in Dictyostelium discoideum . AHQs were extracted with acidified ethyl acetate from supernatants of cultures grown for 18 h in LB-medium. After evaporation of the solvent, the residue was resuspended in 10 ml methanol. Two ml samples were spotted on silica 60F254 TLC plates and developed in a dichloromethane:methanol (95∶5) mixture. Plates were dried and spots visualized under UV-light. PQS and HHQ were undetectable in both the lasR mutant of PAO1 and PT1617 (A). Indicated amounts of D. discoideum cells were deposited on a lawn of P. aeruginosa and incubated at 25°C. The appearance of plaques resulting from growth of the amoeba on bacteria was scored after 10 days. While PAO1 inhibited growth of the amoeba, the lasR mutant and PT1617 were both completely avirulent and permitted growth of amoebal cells (B). (TIF) [file pone.0087814.s002.tif]

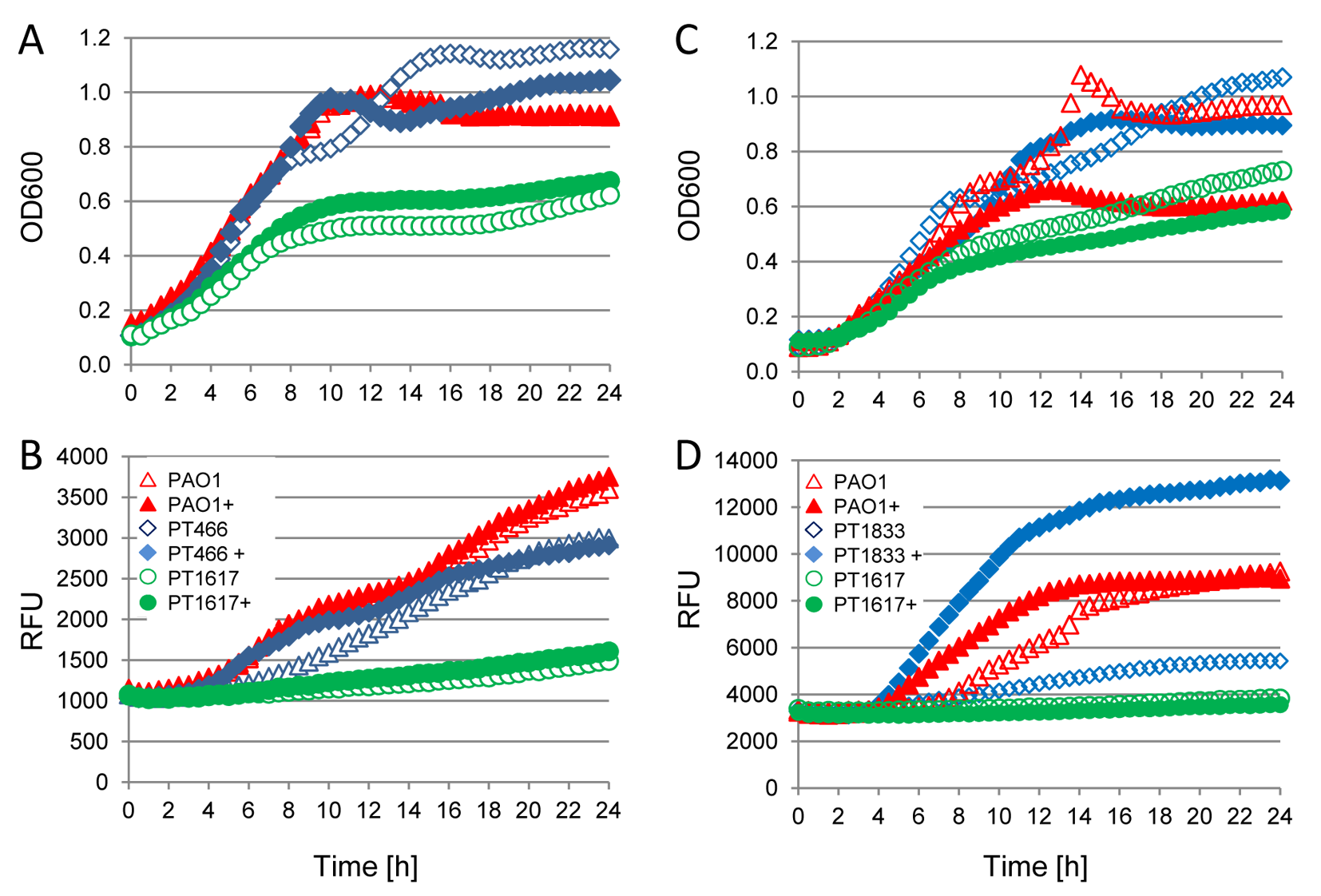

Supplement: Figure S3 — Intracellular detection of externally added 3-oxo-C12-HSL and PQS. 3-oxo-C12-HSL was detected using the lasI-gfp fusion vector pLIGF1 harbored by strains PAO1, a lasI mutant (PT466) and PT1617. PQS was detected using the pqsA-gfp fusion vector ppqsA1 carried by strains PAO1, a pqsA mutant (PT1833) and PT1617. Strains were grown in PB medium supplemented (+) or not with 5 mM 3-oxo-C12-HSL or 50 mM PQS (final concentration) in microtiter plates (200 ml/well). Absorption at 600 nm (A,C) and fluorescence (RFU) (B,D) were monitored for 24 h. Data represent average values of triplicate wells. Standard deviations represented less than 10% of the average value and were omitted for clarity. (TIF) [file pone.0087814.s003.tif]
